# Autoantibodies frequency in children with visceral leishmaniosis

**DOI:** 10.1186/1546-0096-12-S1-P101

**Published:** 2014-09-17

**Authors:** Luciana B Paim-Marques, Raquel Lisboa, Eugenio Pacceli

**Affiliations:** 1 Pediatria, Hospital Dr Waldemar de Alcântara, Brazil; 2 Pediatric rheumathology, Fortaleza University, Fortaleza, Brazil

## Introduction

The visceral leishmaniosis (VL), or Calazar, is a chronic severe systemic disease, potentially fatal to humans. Currently, VL is the prototype of a specific immune dysfunction resulting from parasitism of leishmania donovani in macrophages, producing a broad spectrum of clinical and immunological reversible only with specific treatment. Serum Analysis from infected adult patients demonstrated the presence of autoantibodies against cellular and humoral components, and circulating immune complexes.

## Objectives

To identify the profile of autoantibodies in pediatric patients with VL and its correlation with clinical outcome.

## Methods

Through a transversal study, was investigated the occurence of autoantibodies ( antinuclear antibodies (ANA), anti-DNA , anti-SM , anti-RNP , anti-SSb , anti-SSa, lupus anticoagulant, IgG and IgM anticardiolipin (aCL) antibodies ) in 34 patients (under 18 years) with diagnosis of VL, at the beginning and shortly after treatment, in the period October 2010 to March 2011.

## Results

The incidence of autoantibodies present at the beginning in patients with VL was 64,7% (10 with ANA positive (29,4%), 7 with lupus anticoagulant antibodies positive (20,58%), 8 with IgM aCL antibodies positive (23,5%) and 5 with IgG aCL antibodie positive (14,7%) and 1 with Anti-RNP (2,9%). Sex, age, visceromegaly, nutritional status, treatment, use of corticosteroids, infections, hemophagocytic syndrome, febrile neutropenia, hemoglobin level and platelet count parameters were correlated with the presence of antibodies (table:1). It was found associated anaemia (p<0,05) with the antibody presence, but more studies are needed to evaluate the presence of hemolytic anemia associated. Infections: sepsis, pneumonia and urinary tract infection in 71,42% of total patients, but not correlated with antibodies. Autoimmunity was greatly reduced after treatment; the statistical significance remained after stratification in ANA.

## Conclusion

Visceral leishmaniasis appears to correlate positively with the presence of ANA, lupus anticoagulant, IgG ang IgM aCL, in children, as in adults possibly by triggering a systemic humoral response of Th2. We found association statistically significant with lower hemoglobin level in these patients. Further studies are needed to evaluate the antibodies pattern in these infections.

## Disclosure of interest

None declared.

